# Inflammatory and immunologic analysis of bronchoalveolar lavage from patients with post-COVID-19 lung disease

**DOI:** 10.3389/fmicb.2026.1923299

**Published:** 2026-10-19

**Authors:** Homer L. Twigg, Rachel Wilson, Andy Q. Yu, Wei Li, John A. Altin, Robert D. Tarver, Isabel Knight, Patricia A. Smith, Mark E. Fraser, Peter Castelluccio, Jen Rie

**Affiliations:** 1 Department of Medicine, Indiana University, Indianapolis, IN, United States; 2 Department of Microbiology and Immunology, Indiana University, Indianapolis, IN, United States; 3 Translational Genomics Research Institute, Flagstaff, AZ, United States; 4 Department of Radiology, Indiana University, Indianapolis, IN, United States; 5 Department of Biostatistics and Health Data Science, Indiana University, Indianapolis, IN, United States

**Keywords:** bronchoalveolar lavage (BAL), lung disease, post-COVID, SARS-CoV-2, spike protein

## Abstract

**Rationale:**

Infection with SARS-CoV-2 frequently leads to persistent pulmonary symptoms and radiographic abnormalities, of which several clinical phenotypes have been described. The etiology and inflammatory milieu of these processes is unknown.

**Objectives:**

To study the alveolar milieu in subjects with suspected post-COVID-19 lung disease up to 2 years after acute infection.

**Methods:**

Clinical bronchoalveolar lavage (BAL) samples were processed for analysis of cell differential, presence of spike protein, and SARS-CoV-2 antibody responses. Findings were linked to chest CT findings and clinical outcome.

**Results:**

Clinical BAL fluid samples from patients with suspected post-COVID-19 lung disease can be separated into three inflammatory phenotypes: a lymphocytic predominant pattern, a neutrophilic predominant pattern, and a normal cellular differential. Spike protein is found in BAL many subjects at the time of study. Robust antibody responses to SARS-CoV-2 are seen in many subjects. No clear association was observed between chest CT scan patterns and the type of inflammatory milieu in the lung. BAL inflammatory pattern was unable to predict long term outcome, though early bad outcomes were not seen in subjects with a normal BAL differential.

**Conclusion:**

Suspected post-COVID-19 lung disease exists in several forms, each with characteristic inflammatory milieus. Persistent viral protein may contribute to these responses. These findings highlight the potential difficulty of performing studies of post-COVID-19 lung disease predicated solely on clinical presentation due to patient variability.

## Introduction

Within 6 months following the onset of the COVID pandemic, it was recognized that long-term consequences after resolution of acute disease, termed long COVID or post-acute sequelae of COVID-19 (PASC), was common (Alwan and Johnson, 2021; Xie et al., 2024). In the lung, early reports described long term respiratory symptoms and radiographic changes due to pulmonary fibrotic and inflammatory processes in up to 70% of patients (Caniego-Casas et al., 2022; Han et al., 2021; Lerum et al., 2021; McGroder et al., 2021; Myall et al., 2021; Salvi et al., 2022; Valenzuela et al., 2021). Given the myriad reports describing long COVID in many different organ systems, the NIH sponsored multicenter RECOVER initiative was created to study PASC in all its forms. As a result, large cohorts of patients with PASC have been generated and extensively clinically characterized, including subjects with lung symptoms (Patton et al., 2024), which has been labeled post-COVID-19 lung disease. The RECOVER initiative recognizes The National Academies of Sciences, Engineering, and Medicine (NASEM) definition of long COVID, which states it is “an infection-associated chronic condition (IACC) that occurs after SARS-CoV-2 infection and is present for at least 3 months as a continuous, relapsing and remitting, or progressive disease state that affects one or more organ systems.” Importantly, this is a purely clinical definition and is reliant on the presence of symptoms and other clinical findings such as radiographic abnormalities after a documented SARS-C0V-2 infection. Investigators in the RECOVER initiative and elsewhere are working to further define the underlying inflammatory milieu and cause of long COVID. This is especially important in the lungs, where different inflammatory diseases can have similar clinical and radiographic presentations.

The cause of prolonged pulmonary disease after COVID-19 infection is not known. Outside of autopsy studies there is very limited data on the inflammatory milieu in the lung in patients with suspected post-COVID-19 lung disease. Other viral infections have been associated with pulmonary fibrosis (Huang and Tang, 2021) and cryptogenic organizing pneumonia (COP) (Asai et al., 2017), each of which has characteristic pulmonary inflammatory patterns. What drives the chronic immune and inflammatory response is unclear, but persistent viral antigen stimulation well after acute infection has resolved has been described in many viral infections (Virgin et al., 2009; Choutka et al., 2022), including those with HIV (Chelvanambi et al., 2019; Twigg 3rd., 2018) and influenza virus (Zammit et al., 2006).

Indiana University has a CLIA approved Clinical Bronchoalveolar Lavage (BAL) laboratory used by clinicians seeking to evaluate patients with diffuse lung disease. Since October 2020 we have been receiving BAL fluid specimens from subjects who were being evaluated for post-COVID-19 lung disease. In this work we characterized the alveolar cellular milieu in patients with suspected post-COVID-19 lung disease a median of 178 days after acute infection. We further present preliminary data assessing the pulmonary antibody response after COVID-19 infection. Finally, we describe persistence of SARS-CoV-2 spike protein well after acute infection has resolved and attempt to link these findings to inflammatory and immune responses and long-term clinical outcome.

## Methods

### Sample acquisition

Our CLIA approved BAL laboratory is widely used across central Indiana by clinicians seeking to evaluate patients with diffuse interstitial and alveolar lung disease. Clinically indicated samples are sent to the lab on ice and processed by cytocentrifugation to perform cell differential analysis and lymphocyte phenotyping, when indicated. Starting in December 2002, we obtained IRB approval for store remnant clinical specimens (cells and acellular BAL) for research purposes.

In October 2020, we received our first post COVID-19 clinical bronchoscopy BAL fluid specimen. In 2021, it became apparent there were a lot of patients presenting with persistent radiographic and clinical symptoms well after acute COVID-19, and we started receiving clinical specimens from patients undergoing bronchoscopy as part of their clinical work-up. The only requirement for inclusion in our cohort was a documented positive SARS-CoV-2 PCR at some point prior to their clinical BAL. As of May 5th, 2022 we had obtained 37 BAL samples from 37 different COVID patients. Five were < 28 days of having a known positive SARS-CoV-2 PCR and are considered acute infections. The remaining 32 were obtained between 28 and 730 days after diagnostic PCR. The dates of initial SARS-C0V-2 positivity and last follow up were obtained by chart review. Vaccination and immune status at the time of bronchoscopy were also abstracted from chart review if available. Immunosuppression, when present, was classified as mild, moderate, or severe based on criteria published by Li and colleagues (Li et al., 2023). Follow up data obtained included vital status as of January 1st, 2026 or date of death (whichever occurred first), need for lung transplant, and need for supplemental oxygen. We utilized two control populations. The first consisted of 5 normal volunteers undergoing research bronchoscopy. The second consisted of 7 subjects who had clinical BALs performed but did not have COVID-19 based on the fact they were obtained prior to the pandemic.

### BAL processing

Clinical BALs were performed by the referring physician. BAL fluid was kept on ice until processed as previously described (Twigg III et al., 2008; Wood et al., 2003) to yield cytocentrifugation preparations for determination of cell differential, acellular BAL for cytokine and chemokine analysis, and cell pellets which are stored for future studies. Any specimen that contained greater than 15% buccal mucosal cells and/or airway epithelial cells was considered contaminated and not included in this study. Specimens containing ≥ 15% lymphocytes underwent CD3, CD4, and CD8 phenotyping using methods described by our lab (Smith et al., 2006). After determining BAL cell differentials, all remaining control and suspected post-COVID-19 BALs were aliquoted and stored at −80°. All subsequent experiments were performed on batched samples to minimize testing procedure variability.

### Spike protein antigen detection

SARS-CoV-2 spike protein was measured in acellular BAL fluid using a commercially available BioUltra SARS-CoV-2 Spike Protein ELISA Kit (Kerafast). The sensitivity of the kit is 0.256 pg./mL. The detection range is between 0.256 and 8,000 pg./mL. All samples were run in duplicate. For all 32 subjects with suspected post-COVID lung disease and 10 control subjects, plasma and BAL BUN concentrations were available to determine epithelial lining fluid amounts as described by Rennard and colleagues (Rennard et al., 1985).

### Measurement of anti-SARS-CoV-2 antibody

Anti-SARS-CoV-2 antibodies in BAL fluids from the first 23 subjects with suspected post-COVID-19 lung disease were assessed using a PepSeq platform as previously described (Elko et al., 2022; Henson et al., 2023). This approach enables the rapid generation of fully-customizable libraries containing 1000s-100,000s of DNA-barcoded peptides that can be used to perform highly-multiplexed serological assays, with next generation sequencing read-out. For these studies a human virome-wide assay comprising 15,000 peptides was utilized. PepSeq samples were sequenced to a depth of >50 reads per sample per peptide. Background reactivity was estimated using no-sample (superblock-only) controls. We used the background samples to generate bins within which Z-scores were calculated for each BAL sample, using a method that we described previously (Henson et al., 2023). There was an observed high Z-score concordance between sample replicates (median = 0.94). Neutralizing anti-SARS-CoV-2 antibody activity was measured using the GenScript’s cPass SARS-CoV-2 Neutralizaion Antibody Detection Kit per manufacturer’s instruction (GenScript USA, Inc., Piscataway, NJ). This assay analyzes the ability of biological samples to block binding between spike protein and the ACE2 receptor. BAL fluid was diluted 1:10 with the Sample Dilution Buffer provided in the cPASS kit prior to testing according to the manufacturers recommendation for studies in plasma. The OD450 values for the 1:10 diluted BALF fell within the range of the negative and positive controls from the kit. Duplicated samples had similar OD450 values. A cutoff of 30% signal inhibition is used to define positive detection of neutralizing antibodies for SARS-CoV-2.

### Clinical assessment

All subjects had chest CT scanning prior to bronchoscopy. These scans were reviewed blindly by a NIOSH B Reader radiologist and grouped into predominantly ground glass opacities, predominantly fibrotic or interstitial changes, an equal mixture of both, and other. Long term outcomes were performed on all subjects by chart review as of last documented clinical follow up or January 1, 2026 whichever came first. Outcomes were categorized as death or requiring lung transplant, still on oxygen at time of last follow up, and on no oxygen at the time of last follow up.

### Statistics

Subjects with suspected post-COVID-19 lung disease were divided into three groups based upon published guidelines. The lymphocytic predominant group was defined as having ≥ 15% lymphocytes and greater than the percentage of neutrophils (Meyer et al., 2012). The neutrophilic predominant group was characterized by neutrophils ≥ 10% of the total nucleated cell population and greater than the percentage of lymphocytes (Ryu et al., 2007; Watters et al., 1987). All others were defined as having a normal BAL pattern. No eosinophilic predominant group was present in our cohort. Patients with suspected post COVID lung disease were compared to two types of control subjects as described under sample acquisition for analysis of BAL fluid spike protein and antibody levels. Statistical analyses were conducted with SAS 9.4. Demographic characteristics of groups were compared using the Fisher’s exact test for categorical variables and the Kruskal-Wallis test for continuous variables. Differences in SARS-CoV-2 spike protein levels in acellular BAL and epithelial lining fluid between patients with suspected post-COVID lung disease and the non-COVID control group was assessed using a two-sided Welch’s two-sample t-test. Spike protein concentrations below the limit of detection were assigned the lowest detectable value stated by the ELISA kit (0.256 pg./mL). Logistic regression was used to analyze the association between BAL groups and the clinical outcomes. Clinical outcomes were binarized into good (alive and not on oxygen) versus poor outcomes (on oxygen, need for lung transplant, or death). A joint test for group differences was performed using a likelihood ratio chi-square test. Given the smaller sample size and number of outcome events, the regression analysis was unadjusted. Age and gender are summarized in Table 1. The clinical outcome was also summarized descriptively among participants who survived the first 90 days and had at least 90 days of follow-up after BAL. Associations between the presence of SARS-CoV-2 spike protein in BAL fluid and total or neutralizing antibody positivity were evaluated using two-sided Fisher’s exact tests.

**Table 1:** Subject characteristics and BAL patterns.

Demographic characteristics		Overall (*n* = 37)	Acute (*n* = 5)	Normal Diff (*n* = 7)	Neutrophilic (*n* = 13)	Lymphocytic (*n* = 12)	*p*-value
Age^a^		59 (45, 64)	63 (62, 70)	63 (47, 70)	56 (40, 61)	50.5 (43, 59)	0.0976
Age ≥ 60		16 (43%)	4 (80%)	4 (57.1%)	6 (46.1%)	2 (16.7%)	0.0812
Gender	Female	15 (40.5%)	1 (20%)	3 (42.9%)	4 (30.8%)	7 (58.3%)	0.4473
	Male	22 (59.5%)	4 (80%)	4 (57.1%)	9 (69.2%)	5 (41.7%)	
Race	White	26 (70.3%)	5 (100%5)	3 (42.9%)	10 (76.9%)	8 (66.7%)	0.5804
	Black	5 (13.5%)	0 (0%)	2 (28.6%)	1 (7.7%)	2 (16.7%)	
	Hispanic	5 (13.5%)	0 (0%)	2 (28.6%)	2 (15.4%)	2 (16.7%)	
	Asian	1 (2.7%)	0 (0%)	1 (14.3%)	0 (0%)	0 (0%)	
Days post COVID +^b^		-	7 (0–21)	153 (88–401)	177 (27–425)	169 (28–730)	
BAL Differential^c^							
Alveolar Macrophages		-	50 ± 26	86 ± 12	43 ± 28	46 ± 19	
Lymphocytes		-	27 ± 36	6 ± 5	7 ± 6	44 ± 16	
Neutrophils		-	22 ± 21	4 ± 3	47 ± 29	7 ± 7	
Eosinophils		-	1 ± 1	1 ± 1	3 ± 7	2 ± 2	

a Values are expressed as median (q1, q3) for Age.

b Values are expressed as median (min – max) for days post COVID+.

c Values are expressed as average ± standard deviation.

## Results

### BAL characteristics

Table 1 shows the demographics and BAL fluid cellular characteristics of the 37 COVID-19 subjects included in this study and the median time after their initial SARS-CoV-2 positive test that they were evaluated. Supplementary Tables S1 provides important clinical variables of our cohort at the time of bronchoscopy (vaccination and immune status). Five (13.5%) underwent bronchoscopy less than 28 days after having a known positive SARS-CoV-2 PCR and thus are considered acute infections. The remaining 32 samples were obtained a median of 178 days (range 28 to 730 days) after diagnostic PCR and revealed three patterns. The first was essentially a normal BAL cell differential, with no increase in lymphocytes and minimal if any increase in neutrophils. The second was characterized by neutrophils ≥ 10% of the total nucleated cell population and greater than the percentage of lymphocytes. The third pattern was a lymphocytic predominant pattern characterized by lymphocytes ≥ 15% of the total nucleated cell population and greater than the percentage of neutrophils. Of the 12 patients with elevated lymphocytes, 8 had markedly inverted CD4: CD8 ratios which can be seen in patients with hypersensitivity pneumonitis, nonspecific interstitial pneumonia, chronic viral infections, and COP (Meyer et al., 2012; Costabel et al., 1992). In the two subjects with elevated CD4: CD8 ratios, one had underlying sarcoidosis and the other had hypersensitivity pneumonitis associated with exposure to mycobacterial antigens, both known to cause CD4 predominant lymphocytic alveolitis (Hunninghake and Crystal, 1981; Hanak et al., 2006).

### Detection of SARS-CoV-2 spike protein in BAL

In very early work we used a commercial ELISA kit to measure spike protein in acellular BAL fluids from the first 32 subjects in our cohort with suspected post-COVID-19 lung disease and 12 control subjects (5 BAL fluids from normal volunteers and an additional 7 BAL fluids from patients who had samples sent to our clinical BAL lab but did not have COVID-19). Figure 1A shows spike protein was easily detected in unconcentrated BAL fluids from subjects with suspected post-COVID-19 lung disease compared to controls. Even when corrected for dilution and expressed as pg./ml epithelial lining fluid spike protein was found in significantly greater amounts in BAL from subjects with suspected post-COVI-19 lung disease (Figure 1B). Spike protein was detected up to 627 days after their initial diagnostic SARS-CoV-2 test (Figure 1C). These data support the hypothesis that residual SARS-CoV-2 proteins persist in the lung long after acute infection. We have greatly expanded this data in a submitted manuscript describing persistent spike and nucleocapsid proteins as well as SARS-CoV-2 nucleic acids in BAL fluid from this cohort.

**Figure 1:**
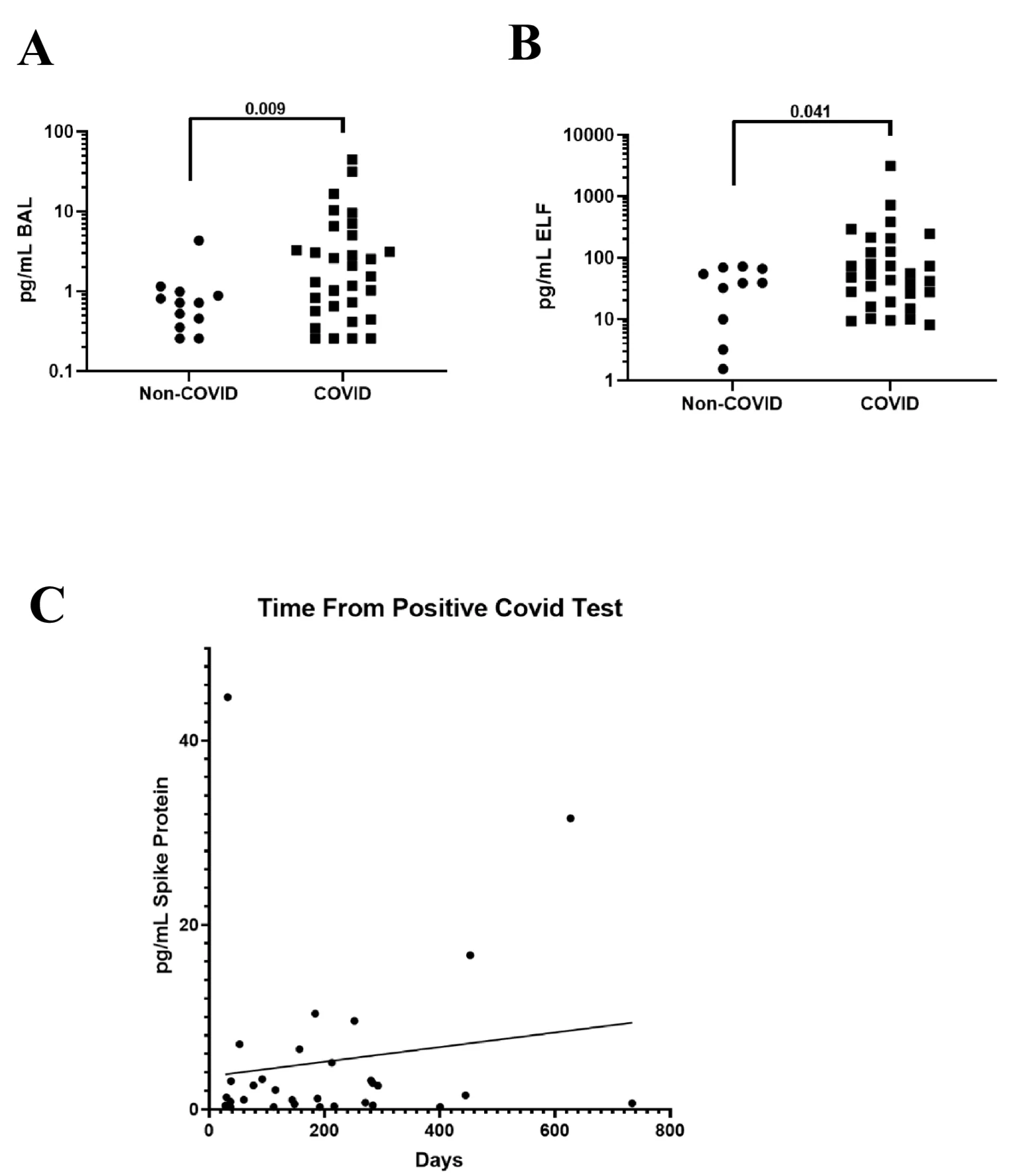
Detection of SARS-CoV-2 spike protein in BAL fluid. Acellular BAL samples from 32 patients with suspected post-COVID-19 lung disease and 12 controls were assayed for spike protein using a commercially available ELISA. **(A)** Spike protein was found in insignificantly greater amounts in BAL samples from patients with suspected post-COVID-19 lung disease compared to controls. **(B)** Similar findings were observed when BAL was corrected for dilution and expressed as pg/ml epithelial lining fluid (ELF). **(C)** The ability to detect spike protein in BAL persisted in some subjects almost 2 years after acute infection.

### Detection of anti-SARS-CoV-2 antibody response in BAL

We next assessed antibody responses in BAL fluids using a previously described PepSeq platform (Elko et al., 2022; Ladner et al., 2021). Figure 2 shows the results from BAL fluid samples from the first 23 subjects in our cohort and 11 controls. Positions of reactive peptides that map to SARS-CoV-2 spike and nucleocapsid protein are shown. Stars indicate the positions of previously described immunodominant linear epitopes, including sites targeted by neutralizing antibodies (Sun et al., 2022; Dacon et al., 2022; Low et al., 2022; Pinto et al., 2021). Fourteen of the 23 (61%) subjects had anti-SARS-CoV-2 antibodies in BAL, including many subjects with a polyclonal response.

**Figure 2:**
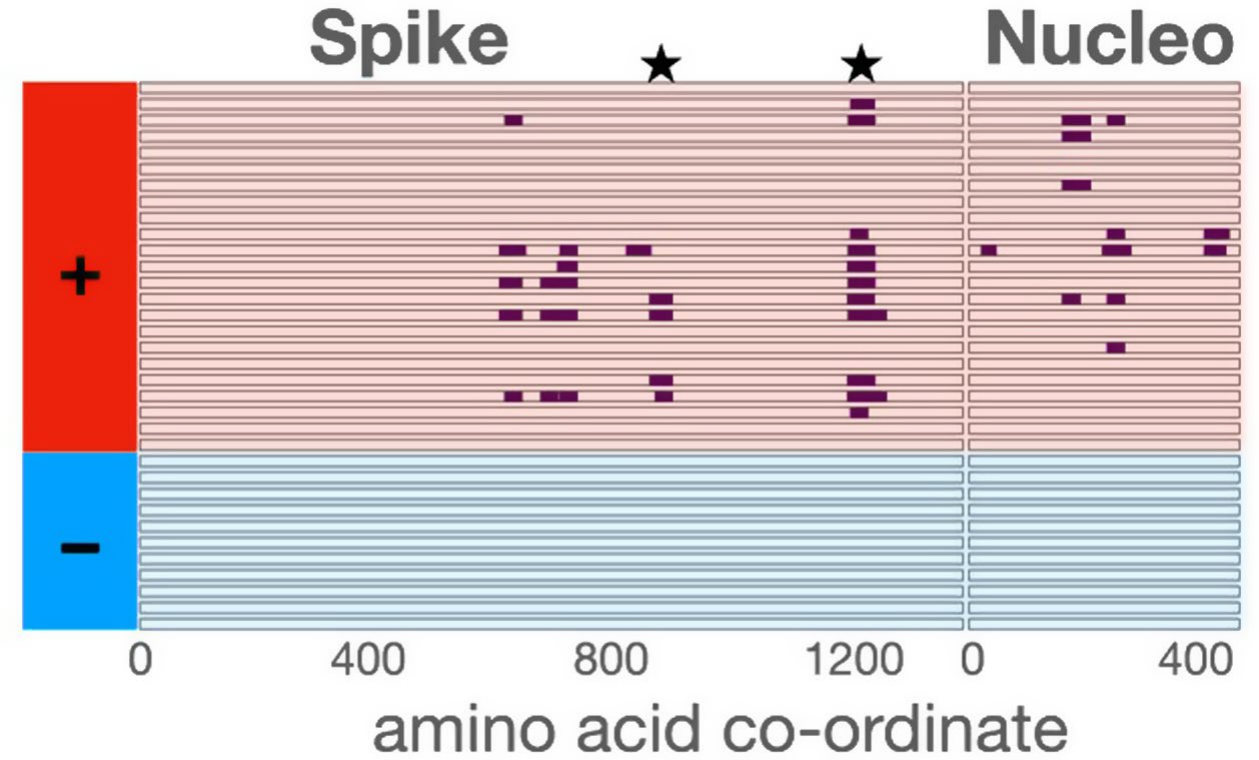
Detection of SARS-CoV-2 antibody reactivity profiles in BAL samples using PepSeq. BAL samples from the first 23 patients with suspected post-COVID-19 lung disease (red) and 11 controls (blue) were assayed for IgG reactivity profiles using a 15,000-plex virome peptide assay. Positions of reactive peptides that map to SARS-CoV-2 Spike (left) and Nucleocapsid (right) are shown. Stars indicate the positions of previously described immunodominant linear epitopes, including site targeted by neutralizing antibodies.

Since the PepSeq library used here consists of unstructured 30mer peptides, it is generally unable to detect reactivity to conformational epitopes. Thus, to assess reactivity we next assessed neutralizing antibody (nAb) activity using the cPass test which tests the ability of biological fluids to block spike protein to the ACE2 receptor (Table 2). Eleven of the 37 (30%) BAL fluid samples from the COVID-19 patients had an inhibition rate above the 30% cutoff set by the assay. Neither healthy BAL samples (*n* = 5) nor non-COVID-19 BAL fluid samples (*n* = 6) had values over 18. There was 100% concordance between the presence of neutralizing antibody and the ability to detect antibodies using the PepSeq assay. As our BALF samples from the healthy controls and non-COVID patients showed a low degree of inhibition, ranging from 6–18%, it was possible that components in BAL fluid could negatively impacted the assay leading to false positives. However, none of the values were above the 30% cutoff. Additionally, none of the COVID samples reached the inhibition level exhibited by the positive control sample from the kit demonstrating that the samples were not under diluted.

**Table 2:** Detection of neutralizing antibody to SARS-CoV-2 in BAL fluid.

Subject sample no.	Percent inhibition	Subject sample no.	Percent inhibition	Control sample no.	Percent inhibition
BAL # 3171	4	BAL # 3151	10	BAL # 140008	10
BAL # 3183	86	BAL #3276	29	BAL # 140027	10
BAL # 3184	0	BAL #3292	0	BAL # 140031	6
BAL # 3188	5	BAL #3295	1	BAL # 140035	8
BAL # 3189	0	BAL #3326	3	BAL # 140039	8
BAL # 3192	0	BAL #3298	79	BAL # 3149	15
BAL # 3213	36	BAL #3315	13	BAL # 3161	14
BAL # 3232	51	BAL #3316	20	BAL # 3253	13
BAL # 3261	11	BAL #3323	34	BAL # 3272	15
BAL # 3263	21	BAL #3333	27	BAL # 3274	18
BAL # 3267	13	BAL #3347	44	BAL # 3287	11
BAL # 3269	12	BAL #3348	41		
BAL # 3257	9	BAL #3352	42		
BAL # 3248	66	BAL #3361	0		
BAL # 3187	10	BAL #3369	11		
BAL # 3219	48	BAL #3372	6		
BAL # 3207	15	BAL #3377	14		
BAL # 3200	27	BAL #3399	79		
BAL # 3146	6				

Yellow = significant inhibition.

### Clinical correlates and outcome

We have performed preliminary comparisons linking BAL fluid patterns and antibody responses to clinical variables. We first examined whether CT findings could predict BAL patterns. Table 3 demonstrates that there was no clear association between the presence of ground glass opacities or interstitial markings (or both) normal, neutrophilic, or lymphocytic BAL patterns. Figure 3 shows the duration of clinical follow up and outcome after BAL. There was no significant correlation between BAL inflammatory patterns and longterm outcome. Interestingly, no early adverse outcomes were observed among subjects with a normal BAL differential, though this study was not powered to establish prognostic differences. The odds ratio of having a good outcome relative to the neutrophilic inflammatory pattern is shown in Table 4.

**Table 3:** Comparison of radiographic patterns of subjects with acute COVID-19 and post-COVID-19 lung disease with different BAL fluid cell differentials.

BAL group	Ground glass opacities	Interstitial infiltrates	Both	Other
Acute (*n* = 5)	3 (60%)	1 (20%)	1 (20%)	
Normal Diff (*n* = 7)	1 (14%)	3 (43%)	2 (28%)	1 (14%)
Neutrophilic (*n* = 13)	5 (38%)	4 (31%)	3 (23%)	1 (8%)
Lymphocytic (*n* = 12)	7 (58%)	2 (17%)	1 (8%)	2 (17%)

**Figure 3:**
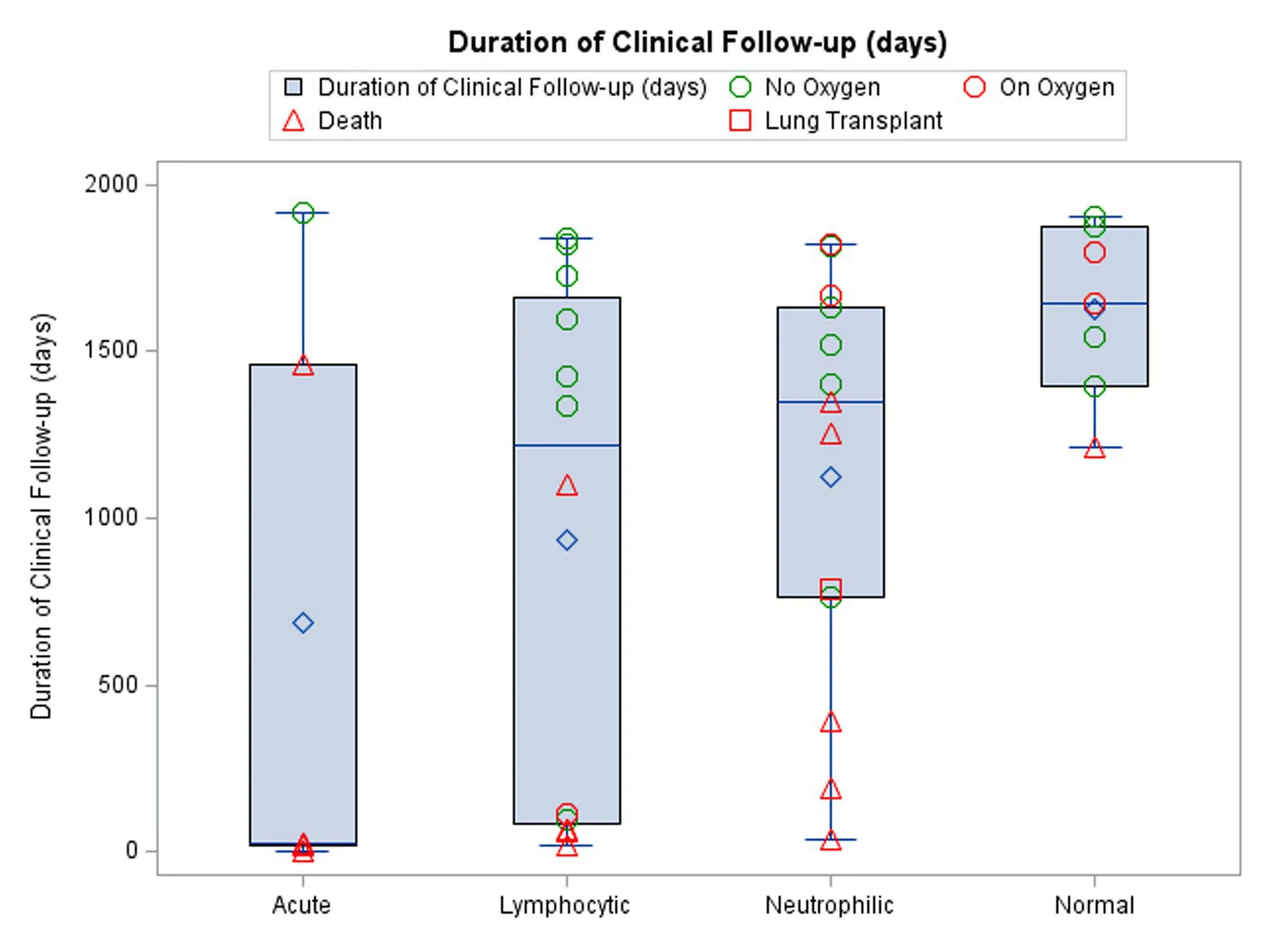
Clinical follow up after BAL. Median duration of follow with first and third quartiles are shown. Poor outcome is defined as death (red triangles) or need for oxygen at time of follow up (red circles). Good outcome is defined as alive and on no oxygen (green circles).

**Table 4:** Comparison of good outcomes between different BAL inflammatory phenotypes.

BAL group	Good outcome, n/N (%)	Unadjusted OR (95% CI)
Acute	1/5 (20.0%)	0.40 (0.03–4.68)
Normal	4/7 (57.1%)	2.13 (0.33–13.81)
Neutrophilic	5/13 (38.5%)	1.00 (Reference)
Lymphocytic	7/12 (58.3%)	2.24 (0.45–11.11)

Finally, we analyzed our preliminary data to assess whether antibody production was associated with spike protein and clinical phenotype. Based on the upper limit of the third quartile of 0.91 pg./mL for spike protein in BAL of normal subjects, we conservatively chose a spike protein in BAL > 2 pg./mL as considered positive. There was no relationship between the presence of spike protein and either total or neutralizing antibody in BAL fluid (Table 5). Five out of five subjects with a normal BAL cellular differential had a detectable total antibody response compared to 25, 56, and 60% of subjects with acute disease, a neutrophilic BAL pattern, or a lymphocytic BAL pattern, respectively (Table 6). Only 14% of subjects with a normal BAL differential had a neutralizing antibody response.

**Table 5:** Relationship between the presence of spike protein in BAL fluid and antibody response in the lung.

Antibody response	Spike positive	Spike negative
Total antibody positive (*n* = 14)	9/13 (69%)	5/10 (50%)
Total antibody negative (*n* = 9)	4/13 (31%)	5/10 (50%)
Neutralizing antibody positive (*n* = 11)	7/18 (37.5%)	4/19 (26.6%)
Neutralizing antibody negative (*n* = 26)	11/18 (62.5%)	15/19 (73.3%)

**Table 6:** Relationship between antibody, spike protein, and clinical classification.

BAL group	Total Ab positive (n = 14/23)	Neutralizing Ab positive (*n* = 11/37)	Spike protein present (*n* = 18/37)
Acute	1/4 (25%)	1/5 (20%)	3 (60%)
Normal	5/5 (100%)	1/7 (14%)	3 (43%)
Neutrophilic	5/9 (56%)	4/13 (31%)	7 (54%)
Lymphocytic	3/5 (60%)	5/12 (46%)	5 (42%)

## Discussion

In this work we have described three bronchoalveolar lavage phenotypes in patients being evaluated for post-COVID-19 lung disease a median 178 days post-acute infection: a bland non-inflammatory cellular differential, a neutrophilic predominant pattern, and a lymphocytic predominant pattern. There is evidence of persistent SARS-CoV-2 spike protein in these patients. Many subjects have broad anti-SARS-CoV-2 antibodies in the lung, including neutralizing antibodies. The radiographic appearance on chest CT is not able to reliably delineate what type of BAL fluid pattern exists. Finally, in preliminary analysis benign BAL fluid patterns do not predict long term outcomes, though subjects with a normal BAL differential did not have early bad outcomes.

Reports describing the persistence of radiographic lung abnormalities on chest CT scanning after COVID-19 infection are plentiful and remarkably consistent. Three early reports showed reticulations consistent with interstitial disease in 35–42%, ground glass opacities in 27–40%, and normal findings in 38% of subjects (Han et al., 2021; Lerum et al., 2021; McGroder et al., 2021). A large publication from the United Kingdom described residual lung abnormalities on chest CT in 79.6% of 209 subjects a median of 119 days after acute infection (Stewart et al., 2022). These four independent reports are remarkably similar in describing residual ground glass opacities and fibrotic changes in up to 80% of patients with COVID-19 pneumonia. Importantly, all these reports described patients with relatively short follow up (≤ 6 months). We describe similar radiographic findings up to 2 years after acute infection.

Little is known about the final clinical and pathologic diagnoses in these patients. One study following 837 patients 4 weeks after discharge revealed 39% had persistent respiratory symptoms and COP was ultimately diagnosed in 35 (4.8%) (Myall et al., 2021). Usual interstitial pneumonia (UIP) is the most common finding in lung biopsies of patients with persistent clinical and radiographic findings after COVID-19 (Konopka et al., 2021). Interestingly, unlike the UIP found in idiopathic pulmonary fibrosis, which usually progresses to death in 3–5 years, many of the UIP fibrotic changes found in patients post COVID-19 infection do not progress, and may even resolve, similar to the experience seen after SARS-CoV-1 (Bazdyrev et al., 2021; Hui et al., 2005). Our results complement this clinical literature in several ways. First, while not providing a definitive diagnosis, patients with a CD8 predominant lymphocytic pattern may have COP and thus have the potential to respond to steroid treatment (Costabel et al., 1992). At a minimum, the presence of a lymphocytic alveolitis is increasingly being recognized as demonstrating the patient has steroid responsive process (Suter et al., 2026). Second, patients with neutrophilic inflammation may have the well described UIP in patients with suspected post-COVID-19 lung disease, though this would require correlation with biopsy and radiographic findings. Third, our patients with normal BAL fluid, who underwent bronchoscopy because of persistent CT findings, likely have a stable scar from their acute infection, which could explain why some patients suspected to have post COVID-19 UIP do not progress and may even resolve.

The cause of prolonged pulmonary disease after COVID-19 infection is not known. However, viral infections, in general, have been clearly associated with pulmonary fibrosis (Huang and Tang, 2021) and COP (Asai et al., 2017). We speculate persistent viral antigen stimulation after acute infection has resolved is driving this response, a hypothesis that was espoused by RECOVER investigators shortly after the discovery of long COVID (Chen et al., 2023). A detailed autopsy studies performed a median of 39 days (9–108) after infection revealed persistent viral genomic RNA in all but one patient (Caniego-Casas et al., 2022). Viral subgenomic RNA, which is present during active SARS-CoV-2 replication, was seen in 65% of patients with disease up to 6 weeks and 33% in those with disease over 6 weeks. Using immunohistochemistry, viral proteins were seen in 3 out of 4 patients whose duration of illness was less than 2 weeks. None was found in 23 patients more than 2 weeks out from infection. More recently, a systematic review of 6 studies demonstrated the presence of SARS-CoV-2 RNA in various tissues (plasma, stool, urine, nasal and oral swabs) in 5–59% of subjects studied up to 6 months after acute infection (Fernandez-de-Las-Penas et al., 2024). Our results add to these investigations by demonstrating persistence of SARS-CoV-2 spike protein in BAL fluid up to 2 years after acute infection. The finding of spike protein in BAL is consistent with other investigators describing persistent viral proteins well after other viral infections (Virgin et al., 2009; Choutka et al., 2022), including HIV infection (Chelvanambi et al., 2019; Twigg 3rd., 2018) and influenza infection (Zammit et al., 2006).

The finding of spike protein in BAL fluid in patients with suspected post-COVID-19 lung disease supports a hypothesis that persistent viral products can lead to chronic lung inflammation. Residual viral antigens can result in persistent stimulation of the immune system leading to chronic inflammation through multiple mechanisms, including persistent B and T cell responses and the development of immunosenescence, a highly pro-inflammatory process resulting from chronic T cell stimulation (Deeks et al., 2012; Kahan et al., 2015). Studies report persistent stimulation of humoral and cellular immunity leading to alterations in B and T cell responses and evidence of cellular exhaustion, a hallmark of immunosenesce (Rha and Shin, 2021; Shuwa et al., 2021). Chronic stimulation of the innate immune system is also likely contributing to the development of long COVID syndromes. SARS-CoV-2 can stimulate macrophages directly through TLR2 to produce inflammatory cytokines (Khan et al., 2021; Zhang et al., 2021). Recent studies have examined the important role of complement activation in patients with long COVID (Notarte et al., 2026). Since viral proteins are known to activate the complement system through innate immune pathways (Stoermer and Morrison, 2011), persistent SARS-CoV-2 proteins could result in chronic neutrophilic inflammation similar that seen in a third of our patients. These considerations are critically important when assessing whether persistent protein expression is beneficial or harmful. While residual viral antigens may lead to persistent SARS-CoV-2-specific responses, ultimately the goal is to clear them completely to avoid persistent immunologic stimulation. It is unknown which immune response is most important for complete viral clearance in the lung, whether it be neutralizing IgG antibody, antibody to specific immunodominant epitopes, mucosal IgA antibody responses (Sterlin et al., 2021), or cellular based responses. In this regard, it is intriguing to note that some form of immunosuppression (as defined in a recent report on SARS-CoV-2 viral dynamics and immune status) (Li et al., 2023) was prevalent in our cohort (28/37 or 76% of subjects), suggesting this may contribute to failure to clear the virus.

There are limitations to our study. First, the cohort size is relatively small and clearly underpowered when attempting to link alveolar inflammatory patterns with clinical variables such as radiographic findings and outcomes. Thus, the prognostic association of clinical findings with BAL phenotype should be considered preliminary pending validation in larger cohorts. Second, these subjects were studied relatively early in the pandemic (last BAL obtained was in May 2022) when SARS-CoV-2 strains were more virulent and prior to wide-spread vaccination. The greater ability of earlier SARS-CoV-2 strains to cause long COVID symptoms has recently been emphasized (Lugtu et al., 2026). Thus, our findings may not apply to patients who have milder forms of acute infection, patients who do not undergo bronchoscopy because of the mildness of their clinical presentation, and patients who have undergone extensive prior vaccination, factors known to affect both short and long-term outcomes after SARS-CoV-2 infection (Talasaz et al., 2023). We also do not have not yet studied a cohort of subjects who had prior SARS-CoV-2 infection but do not have evidence of chronic lung disease. Third, the small sample size precluded adjustment for age, gender, and various clinical conditions including immunosuppression and comorbidities. This includes assessment of pre-existent obstructive lung disease, which has been shown to affect the severity of acute SARS-CoV-2 infection and the development of long COVID (Dalfardi et al., 2026). Finally, the cPass assay 30% cutoff for positivity were developed for serum or plasma and have not been formally validated for BAL fluid. Thus, the neutralization antibody findings should be considered exploratory. All of these limitations limit our ability to link the presence of post-COVID-19 lung disease specifically to residual viral proteins and the presence or absence of specific anti-Sars-CoV-2 antibody responses.

In summary, we have described three different patterns of post-COVID-19 lung disease with different cellular profiles. Evidence of persistent SARS-CoV-2 proteins is seen in patients up to 2 years out from acute COVID-19 infection. Pulmonary humoral responses are easily detectable as well. There was no clear association between different BAL profiles and chest CT imaging patterns. Finally, while different BAL profiles do not have prognostic significance in this small sample size, subjects without evidence of lung inflammation (normal BAL differential) do not exhibit early bad outcomes on follow up. These results demonstrate that post-COVID-19 lung disease exists in several forms and highlight the potential difficulty of performing studies of post-COVID-19 lung disease predicated solely on clinical presentation due to patient variability. Whether residual SARS-CoV-2 components are contributing to these clinical phenotypes and whether SARS-CoV-2 specific immune responses can ameliorate post-COVID-19 lung disease is currently under study.

## Data Availability

The raw data supporting the conclusions of this article will be made available by the authors, without undue reservation.
