# Supplementary material for: Inflammatory and immunologic analysis of bronchoalveolar lavage from patients with post-COVID-19 lung disease
Source: Front Microbiol. 2026 Oct 19;17:1923299. doi: 10.3389/fmicb.2026.1923299 (PMC13724402; doi:10.3389/fmicb.2026.1923299)
Supplement: Supplementary file 1 [file Table_1.docx]

| **Supplemental Table 1. Bronchoalveolar Lavage Pattern, Immune Status, and Vaccination Status in Study Cohort.** | | | | | | | |
| --- | --- | --- | --- | --- | --- | --- | --- |
| **Sample No.** | **BAL Pattern** | **Immune status**  **At Bronch**  **(Reason)** | Vaccination status prior to Bronchoscopy  (#, time from first, time from last) | **Sample No.** | **BAL Pattern** | **Immune Status**  **At Bronch**  **(Reason)** | Vaccination status prior to Bronchoscopy  (#, time from first, time from last) |
| BAL # 3151 | No | Severe Transplant | No | BAL # 3269 | N | Competent | Yes X 2 (91 days after 1st, 58 days after last) |
| BAL # 3146 | A | Severe Lymphoma | No | BAL # 3276 | N | Moderate  pred, benralizumab | Yes X 1 (20 days) |
| BAL # 3171 | No | Mild  DM | No | BAL # 3292 | L | Severe  Leukemia | Yes X 2 (204 days after 1st, 183 days after last) |
| BAL # 3183 | L | Severe  Transplant | No | BAL # 3295 | N | Competent | Yes X 1(141 days) |
| BAL # 3184 | N | Competent | No | BAL # 3298 | L | Severe  Myelodysplasia | Yes X 2 (176 days after 1st, 148 days after last) |
| BAL # 3187 | L | Competent | No | BAL # 3315 | No | Moderate  Carcinoid | Yes X 2 (205 days after 1st, 184 days after last) |
| BAL # 3188 | N | Mild  DM | No | BAL # 3316 | N | Mild  Cirrhosis | No |
| BAL # 3189 | A | Severe  Transplant | No | BAL # 3323 | N | Moderate  Esophageal Cancer | Yes X 3 (265 days after 1st, 33 days after last) |
| BAL # 3192 | N | Mild  DM | No | BAL # 3326 | L | Moderate  ocrelizumab | Yes X 2 (207 days after 1st, 172 days after last) |
| BAL # 3200 | No | Moderate  hydroxychloroquine | Yes (14 days) | BAL # 3333 | L | Competent | No |
| BAL # 3207 | L | Moderate  methotrexate | No | BAL # 3347 | N | Mild  DM | No |
| BAL # 3213 | N | Moderate  pred | Yes (40 days) | BAL # 3348 | L | Competent | Yes X 2 (206 days from 1st, 185 days from last) |
| BAL # 3219 | No | Mild  DM | Yes X 1 (12 days) | BAL # 3352 | A | Severe  Leukemia | Yes X 2 (348 days from 1st, 327 days from last) |
| BAL # 3232 | L | Mild  HIV | Yes X 2 (24 days after 1st, 3 days after last) | BAL # 3361 | L | Moderate  Pred, Mycophenolate | No |
| BAL # 3248 | N | Competent | Yes X 3 (64 days after 1st, 31 days after last) | BAL # 3369 | N | Severe  Transplant | Yes X 3 (359 from 1st, 57 days from last) |
| BAL # 3257 | N | Competent | No | BAL # 3372 | No | Severe  Multiple Myeloma | Yes X 2 (292 days from 1st, 279 days from last) |
| BAL # 3261 | No | Competent | No | BAL # 3377 | L | Severe  Lymphoma | Unknown |
| BAL # 3263 | A | Severe  IVIG | Yes X 2 (73 days after 1st, 39 days after last) | BAL # 3399 | L | Mild  DM | Yes X 3 (366 days from 1st, 216 days from last) |
| BAL # 3267 | A | Mild  DM | No |  | | | |
| Abbreviations: A = Acute Infection, No = Normal BAL Differential, L = Predominantly Lymphocytic BAL Differential, N = Predominantly Neutrophilic BAL Differential, DM = Diabetes Mellitus, Pred = Prednisone, IVIG = Intravenous Immune Globulin. | | | | | | | |
